# New Insights Into Sunflower (*Helianthus annuus* L.) FatA and FatB Thioesterases, Their Regulation, Structure and Distribution

**DOI:** 10.3389/fpls.2018.01496

**Published:** 2018-10-16

**Authors:** Jose A. Aznar-Moreno, Rosario Sánchez, Satinder K. Gidda, Enrique Martínez-Force, Antonio J. Moreno-Pérez, Mónica Venegas Calerón, Rafael Garcés, Robert T. Mullen, Joaquín J. Salas

**Affiliations:** ^1^Department of Biochemistry and Molecular Biophysics, Kansas State University, Manhattan, KS, United States; ^2^Instituto de la Grasa (CSIC), Campus Universitario Pablo de Olavide, Seville, Spain; ^3^Department of Molecular and Cellular Biology, University of Guelph, Guelph, ON, Canada; ^4^Departamento de Genética, Facultad de Biología, Universidad de Sevilla, Seville, Spain

**Keywords:** sunflower, *Helianthus annuus*, acyl-ACP thioesterase, protein location, FatA, FatB

## Abstract

Sunflower seeds (*Helianthus annuus* L.) accumulate large quantities of triacylglycerols (TAG) between 12 and 28 days after flowering (DAF). This is the period of maximal acyl-acyl carrier protein (acyl-ACP) thioesterase activity *in vitro*, the enzymes that terminate the process of *de novo* fatty acid synthesis by catalyzing the hydrolysis of the acyl-ACPs synthesized by fatty acid synthase. Fatty acid thioesterases can be classified into two families with distinct substrate specificities, namely FatA and FatB. Here, some new aspects of these enzymes have been studied, assessing how both enzymes contribute to the acyl composition of sunflower oil, not least through the changes in their expression during the process of seed filling. Moreover, the binding pockets of these enzymes were modeled based on new data from plant thioesterases, revealing important differences in their volume and geometry. Finally, the subcellular location of the two enzymes was evaluated and while both possess an N-terminal plastid transit peptide, only in FatB contains a hydrophobic sequence that could potentially serve as a transmembrane domain. Indeed, using *in vivo* imaging and organelle fractionation, *H. annuus* thioesterases, *Ha*FatA and *Ha*FatB, appear to be differentially localized in the plastid stroma and membrane envelope, respectively. The divergent roles fulfilled by *Ha*FatA and *Ha*FatB in oil biosynthesis are discussed in the light of our data.

## Introduction

In higher plants, *de novo* synthesized fatty acids can be used to produce glycerolipids in the plastid through the activity of plastidial acyltransferases (prokaryotic pathway: Li-Beisson et al., 2010). Alternatively, they can be hydrolyzed from the acyl-carrier protein (ACP) by acyl-ACP thioesterases that release free fatty acids and the free *holo*-ACP protein (Ohlrogge and Jaworski, 1997). Free fatty acids are exported out of the plastids and re-esterified to CoA in order to form the cytosolic acyl-CoA pool (Koo et al., 2004; Zhao et al., 2010; Aznar-Moreno et al., 2014), which can be used for glycerolipid biosynthesis in the endoplasmic reticulum (eukaryotic pathway). Furthermore, a recent study has also reported the export of acyl moieties from thylakoid phosphatidyl glycerol mediated by a specific lipase (Wang et al., 2017).

Thioesterases are key enzymes in oilseeds, playing an essential role in determining the amount and composition of fatty acids that enter the storage lipid pool (Voelker, 1996). They are plastid targeted enzymes encoded by nuclear genes (Yuan et al., 1996; Mayer and Shanklin, 2005) and acyl-ACP thioesterases fall within the group of thioester active enzymes, which are classified into 25 families of which the plant acyl-ACP thioesterases constitute family TE14^1^. Based on sequence alignments, these enzymes have been further classified into two sub-families, called FatA and FatB (Jones et al., 1995). The FatA and FatB enzymes differ in their substrate specificity, with FatA showing high substrate specificity for monounsaturated oleoyl-ACP (Salas and Ohlrogge, 2002; Serrano-Vega et al., 2005; Sánchez-García et al., 2010; Moreno-Pérez et al., 2011; Aznar-Moreno et al., 2016). Within the FatB subfamily, two groups of enzymes can be found that are referred to as FatB1 and FatB2. The former is only found in plant species accumulating short or medium chain fatty acids, like *Cuphea hookeriana* or *Umbellularia californica* (Jones et al., 1995). They are soluble enzymes that display specificity toward short chained acyl-ACPs and have been very important in plant biotechnology (Eccleston et al., 1996; Voelker, 1996). By contrast, FatB2 enzymes are widely distributed among plant species, and they display specificity toward palmitoyl- and stearoyl-ACPs when assayed with their physiological acyl-ACP substrates (Dörmann et al., 2000; Salas and Ohlrogge, 2002; Aznar-Moreno et al., 2016). The role of these enzymes in correct plant development has been demonstrated by reverse genetics (Bonaventure et al., 2003), and they are probably involved in supplying the saturated fatty acids necessary for the synthesis of distinct essential metabolites, such as sphingolipid long chain bases (Chen et al., 2008). Another differential feature of the FatB2 enzymes is the presence of a hydrophobic domain, possible an outside to inside transmembrane helix anchor (Jones et al., 1995; Facciotti and Yuan, 1998). This feature indicates that these enzymes probably differ in their location as well as their substrate specificity, although this has not as yet been supported by specific data regarding their subcellular locations.

An approximation of the secondary and tertiary structure of plant acyl-ACP thioesterases was first achieved through their homology to bacterial thioesterases, the structure of which was determined by XR diffraction (Lawson et al., 1994; Li et al., 2000). These modeling studies showed these enzymes to have two Helix/4-Stranded Sheet hot dog fold domains in the N-terminal end that determined their specificity (Mayer and Shanklin, 2005; Serrano-Vega et al., 2005), consistent with earlier biochemical studies (Salas and Ohlrogge, 2002). Highly conserved catalytic amino acids were also present in the C-terminal domain of the protein. These models were applied to both the FatA and Fat B thioesterases, allowing amino acids involved in the enzyme-substrate interactions to be identified and making it possible to design thioesterase alleles with improved catalytic properties (Mayer and Shanklin, 2007; Moreno-Pérez et al., 2014). More recent studies determined the crystal structure of FatB1 from *Umbellularia californica* (Feng et al., 2017), a thioesterase that specifically acts on C12 substrates. This thioesterase acts as a dimer, its monomers establishing two tandem hot dog folds that are joined by a flexible linker, one close to the C-terminal domain and other close to the N-terminal one. The N-terminal hot dog domain defines the substrate binding pocket, which can be engineered to change the substrate specificity of the enzyme (Feng et al., 2018). This *Uc*FatB1 is more closely related to other plant thioesterases than to any bacterial isoforms and thus, the structure of this enzyme is likely to be a better reference to model FatA and FatB2 enzymes.

In this study, we have analyzed different aspects of the *Helianthus annuus* (*Ha)*FatA and *Ha*FatB thioesterases, addressing the role of these enzymes in sunflower oil biosynthesis by analyzing the oil composition of standard sunflower lines, as well as their kinetics when acting on their natural substrates and the differences in their expression assessed by real-time quantitative PCR (RT-QPCR). Furthermore, to gain insight into the influence of both enzymes on the final composition of sunflower oil, we carried out three dimensional (3D)-structural modeling. The new data regarding the crystal structure of FatB1 made it possible to model the substrate-binding pockets of both *Ha*FatA and *Ha*FatB, which differed in size and geometry in accordance with the different substrate specificities of these enzymes. Finally, we assessed the subcellular localization of both sunflower thioesterases in transiently transformed tobacco suspension-cultured cells via confocal laser-scanning microscopy (CLSM). In doing so, we show that both *Ha*FatA and *Ha*FatB localized to the plastid stroma, although *Ha*FATB also localized to the plastid inner envelope membrane when co-expressed with an inner membrane marker protein. Consistent with these latter results, the association of *Ha*FatB with plastidial membranes was also demonstrated through western blotting of isolated plastid soluble and membrane fractions. The apparent different functions of these two enzymes are discussed in the light of these results.

## Materials and Methods

### Plant Material and Cell Cultures

The standard sunflower line RHA-274 was used as a control in this work, the seeds of which were germinated in wet perlite at 25°C and transferred to a germination chamber for 2 weeks for full seedling development. These seedlings were then moved to growth chambers at 25°C/15°C (day/night) equipped with fertirrigation lines, and they were maintained on a 16 h photoperiod at a photon flux density of 300 μmol m^-2^ s^-1^. Plant tissues at different stages of development (seeds, roots, stems, leaves, and cotyledons) were frozen and stored at -80°C until use.

Suspension-cultured tobacco (*Nicotiana tabacum* L. cv Bright Yellow-2) BY-2 cells (Kato et al., 1972) were grown in darkness at 26°C on a rotary shaker at 130 rpm in modified Murashige–Skoog medium (Sigma, St-Quentin Fallavier, France). BY-2 cells were maintained and then prepared for biolistic bombardment as described previously (Lingard et al., 2008), sub-culturing the cells every 7 days by transferring 1 mL into 50 mL of fresh medium.

### Lipid Extraction and Fat Content

Lipids were extracted following the method of Hara and Radin (1978), and the total fat content of fatty acid methyl esters (FAMEs) and their composition was determined by Gas Chromatography using heptadecanoic acid as the internal standard (Cantisán et al., 2000).

### Plasmid Construction

In this work, the previously cloned sunflower *Ha*FatA (Serrano-Vega et al., 2005) and *Ha*FatB (Serrano-Vega et al., 2003) sequences (GenBank Accessions AY078350 and AJ242915, respectively) were used to design the primers for cloning and RT-QPCR (**Table 1**). The open reading frames of *Ha*FatA and *Ha*FatB were amplified (minus stop codons) using the NheFatA-F/NheFatA-R and NheFatB-F/NheFatB-R primer pairs, respectively (all primers were synthesized by Eurofins MWG Operon, Germany), introducing *Nhe*I restriction sites at the 5′ and 3′ ends. In addition, the *Ha*FatB gene without the hydrophobic, putative membrane association domain (*Ha*FatBΔ) was generated by PCR splicing (Heckman and Pease, 2007). The sequences before and after the hydrophobic sequence were amplified using the NheFatBΔ-F/NheFatBΔ-R and FatBΔ-F/NheFatB-R primers, respectively (stop codon removed). A mixture of both PCR products was used as template to amplify *Ha*FatBΔ with the primers for each end NheFatB-F/NheFatB-R (stop codon removed). The putative hydrophobic sequence was checked according to Krogh et al. (2001), and the resulting PCR products were cloned into pMBL-T (Genaxxon BioScience GmbH, Biberach, Germany) and sequenced (SECUGEN, Madrid, Spain). The plasmids were then digested with *Nhe*I, and the *Nhe*I DNA fragments were ligated into *Nhe*I-digested pUC18/*Nhe*I mGFP and pRTL2/mCherry to generate *Ha*FatA-GFP, *Ha*FatB-GFP, *Ha*FatB-Cherry and *Ha*FatBΔ-Cherry.

**Table 1:** PCR primers used in this work.

Primer name	Sequence^a^
NheFatA-F	5′-**GCTAGC**ATGCTCTCCAGAGGTGTTC-3′
NheFatA-R	5′-**GCTAG**CTTTTTTTGCGGGTTTTTTCC-3′
NheFatB-F	5′-**GCTAG**CATGGTAGCTATGAGTGCTAC-3′
NheFatB-R	5′-**GCTAGC**AACATTTCCAGCAGAGAAGTG-3′
FatBΔ-F	5′-TTTATCAACCAAATGATGCTGGAATGG-3′
FatBΔ-R	5′-CCATTCCAGCATCATTTGGTTGATAAA-3′
FatA_QPCRF	5′-GGTTCTCGAGAGCATCCCAC-3′
FatA_QPCRR	5′-TCACCACTAGCACAACCGTT-3′
FatB_QPCRF	5′-GTGGCGAGATTGTGAAGGGA-3′
FatB_QPCRR	5′-AGTGACAGGCCCAGACATTG-3′
QHaActin-F4	5′-GCTAACAGGGAAAAGATGACT-3′
QHaActin-R4	5′-ACTGGCATAAAGAGAAAGCACG-3′

*^a^Restriction sites are indicated in bold.*

### RT-QPCR Gene Expression Analysis

Total RNA was extracted from different vegetative tissues and the developing seeds were extracted with the Spectrum Plant Total RNA Kit (Sigma-Aldrich, St. Louis, MO, United States) according to the supplier’s instructions. The corresponding cDNAs were synthesized using the Ready-To-Go T-Primed First Strand Kit (Amersham Bioscience, Roosendaal, The Netherlands). The cDNAs obtained were amplified by RT-QPCR using specific primer pairs (**Table 1**: FatA_QPCRF/FatA_QPCRR for *Ha*FatA and FatB_QPCRF/FatB_QPCRR for *Ha*FatB) and SYBR Green I (QuantiTect^®^ SYBR^®^ Green PCR Kit, Qiagen, Crawley, United Kingdom) in a MiniOpticon system to monitor the resulting fluorescence (Bio-Rad). RT-QPCR reactions were performed according to a previous protocol (González-Thuillier et al., 2015) and efficiency curves were derived from sequential dilutions of the cDNAs. The Livack method (Livak and Schmittgen, 2001) was applied to calculate expression relative to the sunflower actin gene *HaACT1* (GenBank Accession Number FJ487620), amplified using specific primers (**Table 1**: HaActF4/HaActR4). Three biological and two analytical replicates were obtained from each sample.

### Homology Modeling of Sunflower Thioesterase Proteins

Homology modeling of the putative *Ha*FatA and *Ha*FatB protein structures was performed with Deepview and the Swiss Model Workspace software (Guex and Peitsch, 1997^2^), using their protein sequences and an available 12:0-ACP thioesterase crystal structure from *Umbellularia californica* as a template (Protein Data Bank Accession 5X04; Feng et al., 2017). *Ha*FatA (89–361) and *Ha*FatB (129–414) peptides were modeled against this template, with sequence identities of 44.6 and 58.1%, respectively. Molecular docking experiments were performed with SwissDock, using palmitic, stearic and oleic acids as substrates (Grosdidier et al., 2011a,b), and I-TASSER^3^ (Yang et al., 2015). Critical residue mapping and visualizations were performed using the UCSF Chimera package (Pettersen et al., 2004).

### Bioinformatics Analysis

Online programs were used to consolidate the predicted sunflower thioesterase subcellular localization and to identify regions that could be disrupted by translational fusion to the green fluorescent protein (GFP) or m-Cherry protein, including TargetP1.1 (Emanuelsson et al., 2000) and ChloroP1.1 (Emanuelsson et al., 1999). Other programs used to identify the putative transmembrane anchorage regions or the transmembrane tendency were TMHMM (Krogh et al., 2001), OCTOPUS (Viklund and Elofsson, 2008), and ProtScale (Gasteiger et al., 2005).

### Cell Transformation and Transient Expression

Transient transformation of tobacco BY-2 cells was performed with 5 μg of plasmid DNA encoding *Ha*FatA-GFP, *Ha*FatB-GFP, *Ha*FatB-Cherry, or *Ha*FatBΔ-Cherry, and 2.5 μg of plasmid DNA encoding either TIC40-RFP, using a Biolistic^®^ PDS-1000/HE particle delivery system (Bio-Rad). The TIC40-RFP construct, consisting of the Arabidopsis 40-kDa component of the translocon at the inner membrane of chloroplasts (Heins et al., 2002) fused to the N terminus of the red fluorescent protein (Dhanoa et al., 2010). Biolistic bombarded BY-2 cells were incubated for 6 h to allow the expression and sorting of the gene products introduced, and to ensure that any potential negative effects due to protein over-expression were diminished. Cells were fixed in 4% (w/v) formaldehyde, permeabilized with 0.01% (w/v) pectolyase Y-23 (Kyowa Chemical Products, Osaka, Japan) and then permeabilized with 0.3% Triton X-100 (v/v: Sigma-Aldrich). When necessary, the fixed and permeabilized cells were processed for immunofluorescence microscopy, first incubating the BY-2 cells with a rabbit anti-*Arabidopsis* N-acetyl glutamate kinase (NAGK) antibody (Chen et al., 2006) and then, with a fluorescent dye-conjugated secondary antibody: goat anti-rabbit Alexa 488 (Invitrogen) for green emission and goat anti-rabbit rhodamine RedX (Jackson ImmunoResearch Laboratories, Inc., West Grove, PA, United States) for red emission.

### Subcellular Localization of *Ha*FatA and *Ha*FatB

Fluorescence microscopy images of BY-2 cells were obtained on a Leica DM RBE CLSM using a 63× Plan Apochromat oil-immersion objective, a TCS SP2 scanning head and the TCN NT software package (version 2.61: Leica). Samples were excited with an argon laser at 488 nm (GFP) and 543 nm (Cherry, RFP, rhodamine RedX), and fluorescent emission was collected at 500–530 nm for GFP, or at 590–640 nm for RFP, cherry and rhodamine RedX. Fluorophore emissions were collected sequentially in double-labeling experiments, while single labeling experiments exhibited no detectable crossover at the settings used for data collection. Confocal images were acquired as a z-series of representative cells and single optical sections that were saved as 512 pixel × 512 pixel digital images. All fluorescence images shown in the figures are representative of >20 cells from at least three independent transformation experiments.

### Plastid Isolation From Sunflower Seeds

Plastids were isolated from seeds harvested at 15–18 DAF according to the protocol of Jain et al. (2008), with minor modifications. Sunflower seeds (5–10 g) were kept on ice in a plastid isolation medium (PIM: 0.5 M sorbitol, 20 mM HEPES/NaOH [pH 7.4], 10 mM KCl, 1 mM MgCl_2_, 1 mM EDTA, 10% (v/v) ethanediol, 5 mM DTT) and they were homogenized by chopping with a razor blade. The sample was filtered into a fresh tube on ice through one layer of miracloth (25 μm: Calbiochem, United States and Canada) and the filtrates were then centrifuged at 750 g for 5 min at 4°C, recovering the pellets in 2 mL of cold PIM. The material was layered onto 10 mL of 35% Percoll and 3% (w/v) PEG-4000 and centrifuged at 1000 ×*g* for 8 min at 4°C. The upper band (1 cm from the surface) and the pellet that contained the plastids, were gently washed in 10 mL of PIM and centrifuged again at 750 g for 5 min at 4°C. The pellet was again recovered in 400 μL PIM. Pyrophosphate-dependent phosphofructokinase activity was used as cytosolic marker (according to Kang and Rawsthorne, 1996), and the plastids were then lysed by applying five freeze/thaw cycles using liquid nitrogen and water at 50°C. We recovered 100 μL of the lysed plastid sample and isolated the stromal fraction by centrifugation at 100,000 g for 1 h at 4°C. The pellet containing the membrane fraction was resuspended in 300 μL of PIM and the protein concentration in these fractions was determined by the Bradford (1976) method. Proteins from the stromal and membrane fractions (10 μg) were resolved by sodium dodecyl sulphate polyacrylamide gel electrophoresis (SDS–PAGE) and the 10% polyacrylamide gel was then stained with Coomassie™ Brilliant Blue G (Sigma-Aldrich, Germany) or analyzed by Western blotting (Amersham, United States: see Xiao et al., 2008).

### Western Blotting of Plastid Fraction

Proteins were transferred electrophoretically from SDS–PAGE gels to PVDF membranes using the Trans-Blot^®^ Turbo™ Mini Transfer Pack (Bio-Rad, United States). The membranes were then blocked for 1 h at room temperature with 1% non-fat milk in PBS (140 mM NaCl, 2.7 mM KCl, 10 mM Na_2_HPO_4_, 1.8 mM KH_2_PO_4_, [pH 7.4]), washed three times for 10 min with TPBS (PBS plus 0.1% Tween 20) and probed for 2 h at room temperature with the anti-long chain acyl-ACP thioesterase 3-2 antibody (1:5,000 in TPBS; Dörmann et al., 1995). The membrane was washed gently three times for 10 min with TPBS buffer and then antibody binding was detected with a goat anti-rabbit IgG peroxidase conjugated antibody (1:10,000 in TPBS: Thermo Scientific, United States) at room temperature for 1 h. The membrane was washed again three times with TPBS and the protein bands were then visualized using the ECL western blotting detection kit (GE Healthcare), following the manufacturer’s instructions. Images were recorded in a Chemi Genius^2^ Bio-Imaging System (Syngene, India).

### Biochemistry Control Assays

The long chain acyl-CoA synthase (LACS) reaction mixtures contained 100 mM Bis-tris-propane [pH 7.6], 10 mM MgCl_2_, 5 mM ATP and 2.5 mM DTT, and with the CoA [1-^14^C]oleoyl substrate ranging from 0.02 to 0.08 nmol (30–170 Bq approximately). The reactions were started by adding the total, soluble or membrane fraction of the plastid preparation containing approximately 0.5 μg of protein (Aznar-Moreno et al., 2014). The assays were carried out at room temperature for 30 min and then stopped by adding 100 μL of 10% (v/v) acetic acid in isopropanol. The [1-^14^C]oleoyl-CoA was washed three times with 900 μL Hexane:isopropanol:water (50:25:25) and analyzed in a calibrated liquid scintillation counter (LS6500, Beckman Coulter, United States).

Pyrophosphate-dependent phosphofructokinase (PFP) activity was assayed in the reverse direction. The reaction mixture contained 0.1 mM Fru-1,6-diphosphate, 2.5 mM MgCl2, 0.5 mM NADP, 100 mM Hepes buffer pH 8.0, 9U phosphoglucose isomerase, 2U glucose-6-phosphate dehydrogenase in a final volume of 1 mL. A suitable amount of enzyme solution was added to the mixture and incubated for 1–8 min as described above, then reaction was started by the addition of 1 mM orthophosphate and the absorbance at 340 nm was monitored in continuous to measure activity.

Thioesterase activity was assayed in 0.1 ml reactions containing 50 mM Tris-HCl [pH 8.0], 5 mM DTT and [1-^14^C]oleoyl-ACP substrate ranging from 0.02 to 0.08 nmol (30–170 Bq approximately). The oleoyl-ACP was synthetized as described previously (Aznar-Moreno et al., 2016) and the reactions were started by adding of 0.5 μg of the protein preparation (Serrano-Vega et al., 2005). The reactions were carried out at room temperature over 5 min and stopped by adding 0.25 ml of 1 M acetic acid in 2-propanol. Unesterified [1-^14^C]oleoyl was then extracted twice with 0.3 ml hexane and the radioactivity in the pooled organic phase was determined in a calibrated liquid scintillation counter (LS6500, Beckman Coulter, United States).

## Results And Discussion

### Tissue Expression Profiles of Sunflower Thioesterases

**FIGURE 1:**
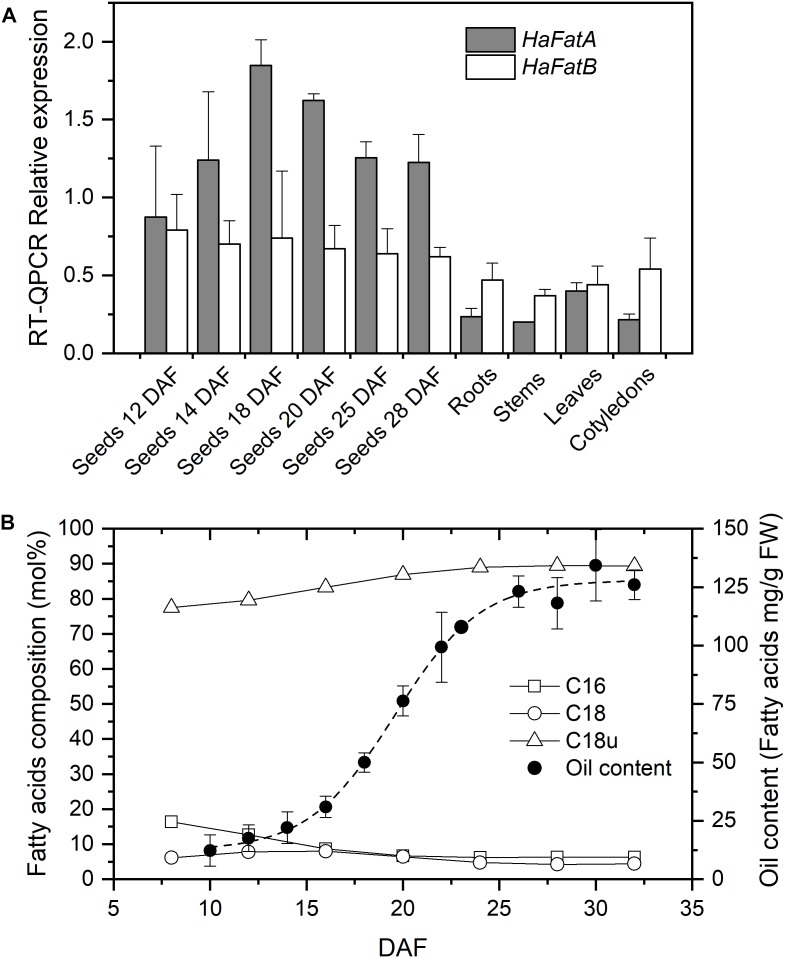
Sunflower acyl-acyl carrier protein (acyl-ACP) thioesterases expression levels and oil composition. **(A)** Relative mRNA expression (RT-QPCR) of *Ha*FatA and *Ha*FatB in sunflower seeds at different developmental stages and in vegetative tissues. The bars corresponded to the SD calculated using the Livak method from three biological and two analytical replicates. **(B)** Fatty acid composition (mol %) and oil deposition (expressed as mg of fatty acid per g FW) during seed development. DAF, Days after flowering; C16, saturates of 16 carbons (palmitic acid content); C18, saturates of 18 carbons and more (the sum of stearic, arachidic, and behenic acids); and C18u, unsaturated of 18 carbons (the sum of oleic and linoleic acids). Dashed line corresponds to the Boltzmann fit of oil content experimental data from 20 seeds per DAF. Standard deviations for fatty acid composition, being smaller than 3% than the average value, are not shown for figure clarity

The expression of the *HaFatA* and *HaFatB* genes in seeds at different developmental stages (from 12 to 28 DAF) and in vegetative tissues (roots, stems, cotyledons and leaves of 20-day-old seedlings) was analyzed by RT-QPCR (**Figure 1A**). Both sunflower genes were expressed most strongly in seeds, particularly *HaFatA*. During seed development, the expression of *HaFatA* doubled in intensity from 12 to 18 DAF, thereafter falling from 20 to 28 DAF. The expression of *HaFatB* remained more or less constant, or with a small reduction from 12 to 28 DAF (**Figure 1A**). *HaFatB* was always the more strongly expressed in vegetative tissues, except for leaves. In this period, the total amount of lipids increased eightfold (**Figure 1B**), with a maximum rate of fatty acid synthesis from 15 to 25 DAF, where the maximum levels of *HaFatA* coincide with an increase in oleic acid synthesis.

The pattern observed here suggests that sunflower FatA acyl-ACP thioesterase is important not only for oil deposition in the seed but also, for the final oil composition. Sunflower oil is rich in unsaturated fatty acids derived from oleate released mainly via FatA (**Figure 1B**) and thus, the synthesis of sunflower oil requires this fatty acid to be exported from plastids at a high rate. This is consistent with the strong *HaFatA* expression during the oil deposition period (10–30 DAF, see **Figure 1B**). The expression of *HaFatB* was maintained during the period of oil accumulation, although it was not as tightly regulated as that of *HaFatA*. This profile of FatA and FatB in developing sunflower oil seeds was very similar to that observed previously in Arabidopsis (Sánchez-García et al., 2010) and *Camelina sativa* (Rodríguez-Rodríguez et al., 2014). Both these species express more FatA than FatB, at least one order of magnitude more in the period of oil accumulation, pointing to an important role of FatA in the export of the fatty acids that accumulate in the oil. A different profile was observed in castor seeds (Sánchez-García et al., 2010), where FatB expression was similar to that of FatA. However, this species has many peculiarities in terms of its oil synthesis, specializing in the accumulation of large amounts of a single fatty acid in its oil. Thioesterases of the FatB type are mainly related to palmitic and stearic acid export from plastids (Bonaventure et al., 2003). Sunflower oil accumulates small amounts of saturated fatty acids, so the role of *Ha*FatB must be related to the supply of saturated acyl chains destined to maintain the correct balance of fatty acids in the plant membranes and that serve as substrates for other biosynthetic pathways. This fact was supported by the high specificity of *Ha*FatB toward saturated fatty acids (Aznar-Moreno et al., 2016) and the decrease in the flux observed in sunflower mutants with a high-saturated oil phenotype (Aznar-Moreno et al., 2014). Furthermore, the relationship between the FatA and FatB thioesterases in providing the necessary activity to supply fatty acids according to the experimental data (**Figure 1B**) assuming similar substrate availability would be 100:1 for oleate export. This ratio cannot be explained through the expression of these enzymes (**Figure 1A**), indicating limited substrate availability for the FatB thioesterase or distinct interactions between this enzyme and the other enzymes involved in fatty acid synthesis. This issue could be influenced particularly by the membrane location of FatB, which could affect the metabolic channeling in conjunction with the soluble fatty acid synthase (FAS; Martínez-Force and Garcés, 2002).

### Tertiary Structure Prediction of *Ha*FAT Proteins and Activity

*Ha*FatA and *Ha*FatB display important differences in their kinetic parameters for the different acyl-ACP substrates present in plant plastids. Preliminary studies of the substrate specificity and catalytic efficiency of these enzymes indicated that *Ha*FatA is likely to be specific for the hydrolysis of oleoyl-ACP, whereas *Ha*FatB displayed a broad specificity profile, displaying similar activity toward oleoyl-, palmitoyl-, and stearoyl-ACP derivatives (Serrano-Vega et al., 2005; Moreno-Pérez et al., 2014). However, later studies demonstrated that *Ha*FatB displayed a highly specificity profile toward saturated acyl-ACP derivatives (Aznar-Moreno et al., 2016). Hence, the plastidial export of oleate and of saturated fatty acids (palmitate and stearate) probably takes place independently, mediated by the action of the two different thioesterases. The expression of these two enzymes (**Figure 1**) was also consistent with this hypothesis. The differences in the substrate specificity of these enzymes appears to be driven by their N-terminal domain (Salas and Ohlrogge, 2002), although the structural traits and specific amino acid residues that determine such differences in specificity remain to be determined.

**FIGURE 2:**
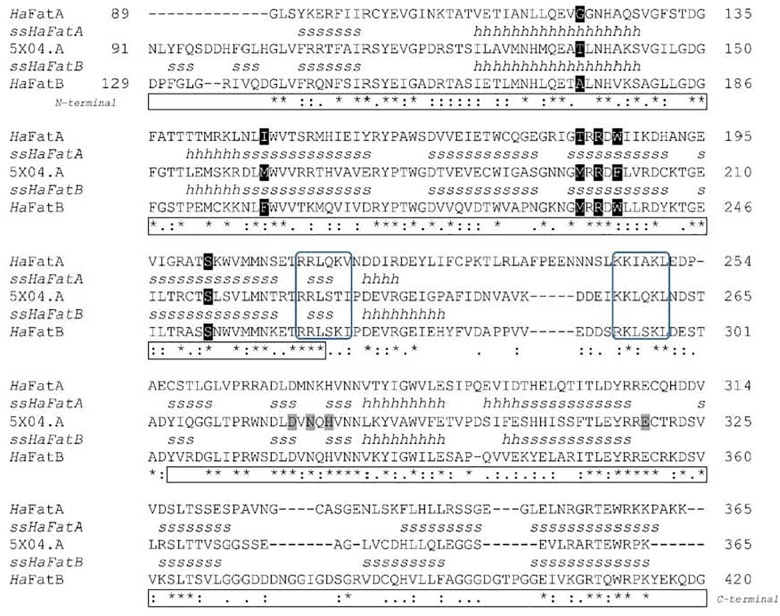
Comparison between the deduced amino acid sequences (*Ha*FatA, *Ha*FatB, and Umbelullaria californica UcFatB) and predicted secondary structures ssHaFatA and ssHaFatB. Asterisks designate identical residues, a colon indicates conservative changes and a dot the weakly conserved changes between the sequences. Structural elements: H, α-helix; S, β-sheet. Residues involved in the substrate binding pocket and catalysis (Feng et al., 2017) are highlighted in black and gray, respectively. Positively charged surface patches (Serrano-Vega et al., 2005; Jing et al., 2018), and the N- and C-terminal domains are boxed in blue and black, respectively.

**FIGURE 3:**
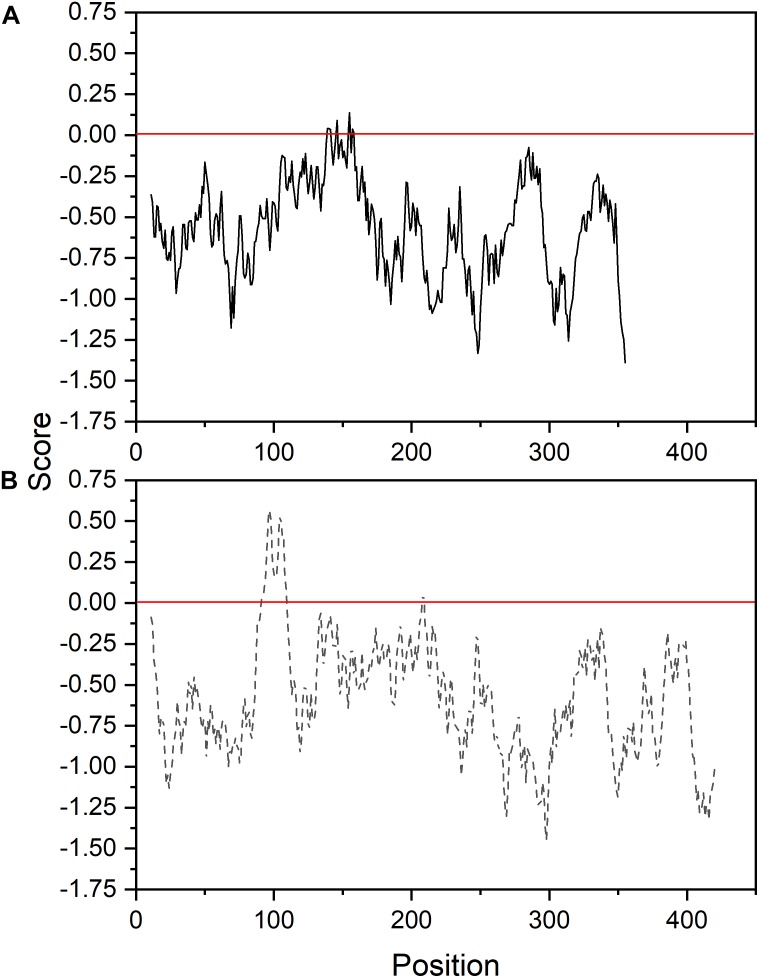
Membrane tendency profile of sunflower FatA **(A)** and FatB **(B)** thioesterases.

The ORF of *Ha*FatB cloned in this work encodes a predicted protein of 430 amino acids, which corresponds to a calculated molecular mass of 47.64 kDa with a pI value of 7.1. Like *Ha*FatA, the first 60 amino acids of the N-terminal domain were predicted to be a transit peptide (**Figure 2**) and thus, L90 was considered to be the first amino acid of the mature *Ha*FatB protein, as proposed for many other plants (Huynh et al., 2002; Jha et al., 2006; Ghosh et al., 2007). *Ha*FatB has a hydrophobic domain from L90 to Q109 (**Figure 3**) that is absent from FatA thioesterases, and it could be involved in membrane anchorage but is not related to the catalytic properties of the enzyme (Jones et al., 1995; Facciotti and Yuan, 1998; Salas and Ohlrogge, 2002). Furthermore, it maintained strong homology to other FatB enzymes in the domain containing the three residues that represent the papain-like catalytic triad required for this activity (Mayer and Shanklin, 2005).

Structural models have been proposed for *Ha*FatA (Serrano-Vega et al., 2005), and for FatB from *Arabidopsis thaliana* and *Cuphea viscosissima* (Mayer and Shanklin, 2005; Jing et al., 2018), and more recently, the crystal structure of FatB1 from *Umbellularia californica* was determined by X-ray diffraction (PDB 5X04.A: Feng et al., 2017). The amino acid sequence, excluding the signal peptide region, of this *Uc*FatB was compared to the *Ha*FatA (residues 89–361) and *Ha*FatB (residues 129–414) sequences, showing 44.57 and 58.05% identity, respectively (**Figure 2**). Thus, the only known plant acyl-ACP thioesterase structure was used as model for these sunflower proteins. As shown in the sequence alignment and through the secondary protein structure, there was 32.5% identity and a high degree of conservation (24%) in the remaining residues. Strong variability was found in the flexible linker located between the N-terminal and C-terminal domains that are involved in substrate specificity and catalysis. The secondary structures of the enzymes were also pretty similar, conserving the catalytic residues (D270, N272, H274, and E308 in *Ha*FatA; and D317, N319, H321 and E354 in *Ha*FatB), although the N-terminal domain shows the highest divergence, the region where the residues conforming the substrate binding pocket can be found. This weaker conservation was possible responsible for the differences in substrate specificity. Indeed, the positively charged surface patches involved in the interaction with ACP (Serrano-Vega et al., 2005; Jing et al., 2018), and that surround the substrate binding pocket, were also well conserved (263-RRLSKI-268 292-RKLSKL-297 in *Ha*FatA; and 212-RRLQKV-217 246-KKIAKL-251 in *Ha*FatB).

**FIGURE 4:**
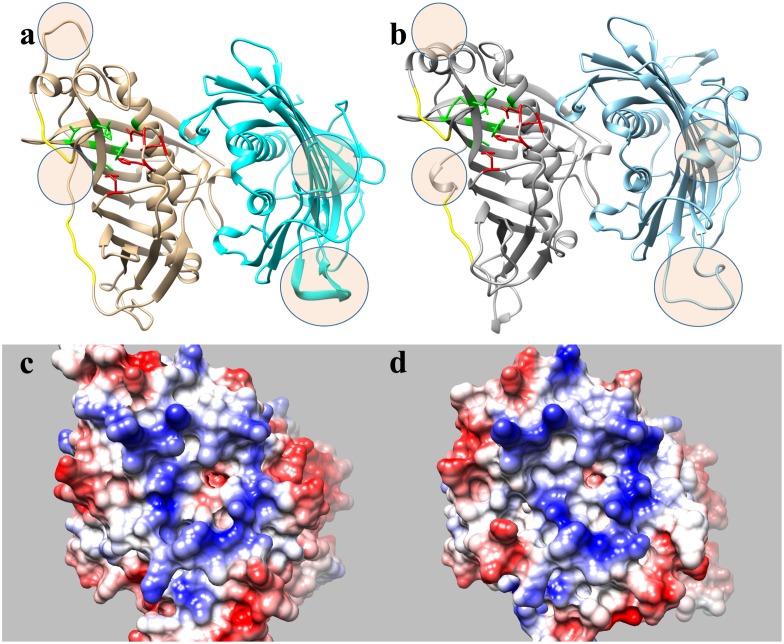
Proposed structural models for sunflower acyl-ACP thioesterase homodimers, *Ha*FatA **(a,c)** and *Ha*FatB **(b,d)**, modeled from UcFatB (5X04.A) (Feng et al., 2017). Ribbon diagrams **(a,b)**, residues in the substrate binding pocket are green and those involved in catalysis are in red. In **c,d**, the molecular surfaces show the electrostatic potential according to Coulomb’s laws with positive charged patches surrounding the substrate binding pocket. Circled areas in **a,b** correspond to highly no conserved structural motifs between both proteins.

**FIGURE 5:**
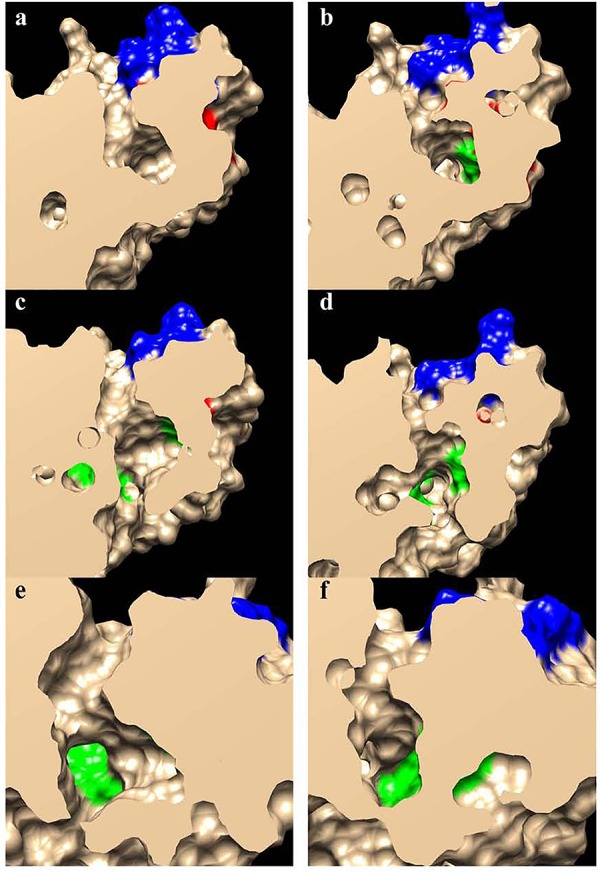
Slab views of the substrate binding pockets for *Ha*FatA **(a,c,e)** and *Ha*FatB **(b,d,f)**. The residues involved in the substrate interaction described by Feng et al. (2017) are shown in green, those involved in catalysis are in red and those involved in the positively charged surface patches surrounding the opening of the pocket are in blue.

The formulation of three-dimensional models (**Figure 4**) allows the structural differences of these proteins to be better understood, particularly since the general structure of these enzymes appeared to be conserved (**Figures 4a,b**). The external region involved in the interaction of the enzymes with the acidic ACP protein had a very similar shape and positive charge, indicating that the main differences between these enzymes probably lies in the binding pocket (**Figures 4c,d**). A detailed view of the substrate channel shows that *Ha*FatB has a tighter gate and less volume (18% less, calculated from volume data for narrow cavities obtained with the PDB viewer program), which would make the interaction with voluminous unsaturated fatty acids difficult (**Figure 5**). By contrast, *Ha*FatA displayed a longer and curved hydrophobic cavity that would interact much better with longer and unsaturated fatty acids.

**FIGURE 6:**
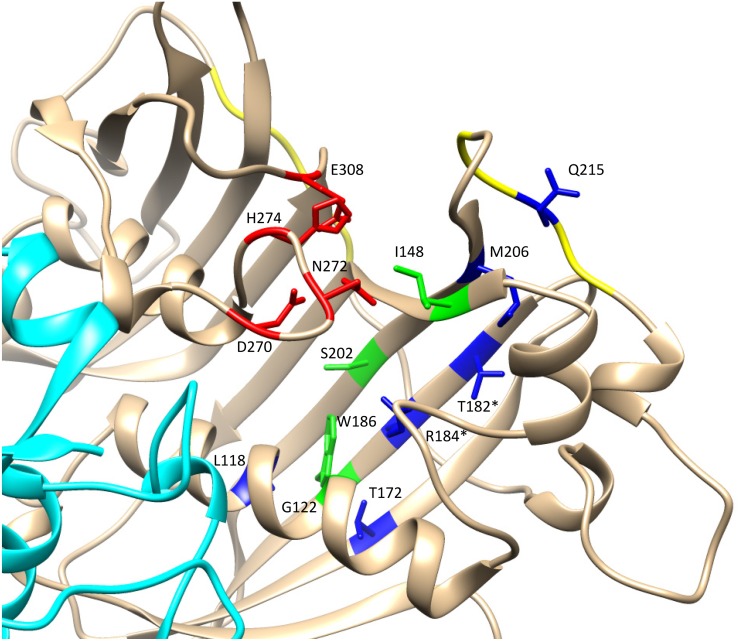
Detail of the catalytic domain in the modeled *Ha*FatA enzyme. Catalytic amino acids are shown in red and amino acids conforming the hydrophobic pocket in green. The amino acids that altered activity when they were substituted by tryptophan (W) were labeled in blue (Moreno-Pérez et al., 2014). When there was a coincidence between the residues modified by mutagenesis and those described by Feng et al. (2017) they are indicated with an asterisk.

The 3D structural data for *Ha*FatA provided details of the position of the residues that affected activity in previous site-directed mutagenesis studies (Moreno-Pérez et al., 2014). In that study, four changes were demonstrated to significantly affect activity: L118W, T182W, M206W and Q215W. In all cases, the original amino acids were changed for a hydrophobic and voluminous tryptophan in order to alter the hydrophobic pocket. The changes L118W and T182W induced an important decrease in the activity, with both residues lying close to amino acids W186 and I148 that conformed hydrophobic pocket of the enzyme (**Figure 6**), suggesting that they probably hampered the correct assembly of the acyl residue within it. Conversely, the amino acid changes Q215W and M206W increased the catalytic efficiency of the enzyme by 2.4 and 3.5-fold, respectively. Interestingly, both amino acids were close to the entrance of the hydrophobic pocket and thus, they probably increase the hydrophobicity of this region, facilitating the interaction between the enzyme and its substrate (**Figure 5**). The M206W change also enhanced the substrate specificity for 18:1-ACP (Moreno-Pérez et al., 2014) and the transient expression of this allele made important modifications to the TAG content of tobacco leaves possible. In *Ha*FatB those residues position corresponded to L169/M233/M257/S266, being Q215/S266 the most conservative change. The influence of these residues in the activity of this enzyme have not been studied yet. Therefore, this new model of plant thioesterase structure could represent a useful tool to engineer these enzymes, providing important information about the amino acids that determine their activity and specificity (Feng et al., 2018).

### Prediction of Subcellular Localization

As a intial step to investigate the subcellular localization of the *Ha*FatA and *Ha*FatB proteins, their deduced amino acid sequences were assessed using several web-based protein location prediction programs, including ChloroP^4^ and SignalP^5^. Overall, both proteins were assigned a high probability of plastid localization. That is, as described previously for plant acyl-ACP thioesterases in general (Mayer and Shanklin, 2005), both *Ha*FATA and *Ha*FATB were strongly predicted to contain N-terminal plastid transit peptide (**Figure 2**), indicating that they target to plastids in plant cells. Notably, *Ha*FatB also contains a distinct hydrophobic sequence that is absent in *Ha*FATA (**Figures 2, 3**) and may serve to anchor *Ha*FATB in (or associate with) a plastid membrane(s) (Yuan et al., 1996; Facciotti and Yuan, 1998; Mayer and Shanklin, 2005).

### Subcellular Localization of *Ha*FATA and *Ha*FATB in Tobacco BY-2 Cells

**FIGURE 7:**
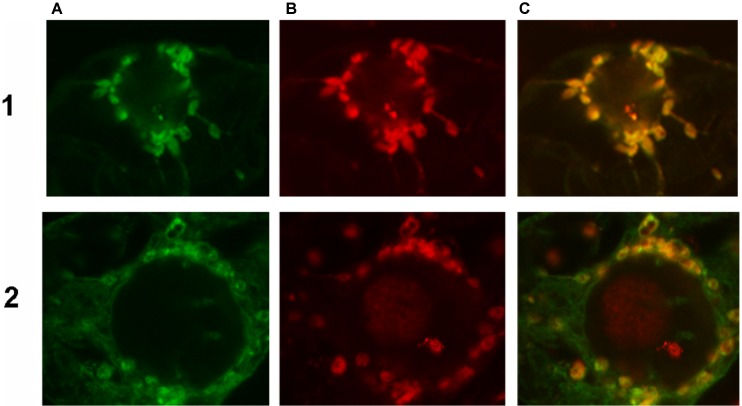
Representative confocal laser scanning microscopy images of green fluorescent protein (GFP)-tagged *Ha*FatA or *Ha*FatB expressed transiently (via biolistic bombardment) in BY-2 tobacco cells. Images in column **(A)** show FatA-GFP row **(1)** and FatB-GFP row **(2)**, while those in column **(B)** represent the fluorescent rhodamine Red dye-conjugated to Anti-N-acetyl glutamate kinase (Anti NAGK). Corresponding merged images of all the cells are shown in column **(C)**.

To confirm the abovementioned predictions, both *Ha*FATA and *Ha*FATB were fused to the GFP, and the resulting fusion proteins were transiently expressed in tobacco BY-2 suspension-cultured cells, which are a well known model system for studying plant protein localization *in vivo* (Brandizzi et al., 2003; Miao and Jiang, 2007). The subcellular distribution of the expressed *Ha*FATA/B-GFP fusion proteins was then assessed (via CLSM) by comparing with the localization of immunostained, endogenous NAGK, which is a plastid stromal protein (Chen et al., 2006). As shown in **Figures 7-1,2**, both transiently expressed *Ha*FATA-GFP and *Ha*FATB-GFP displayed similar fluorescence patterns in BY-2 cells that co-localized with the fluorescence pattern attributable to endogenous NAGK, indicating both proteins are localized in the plastid stroma.

**FIGURE 8:**
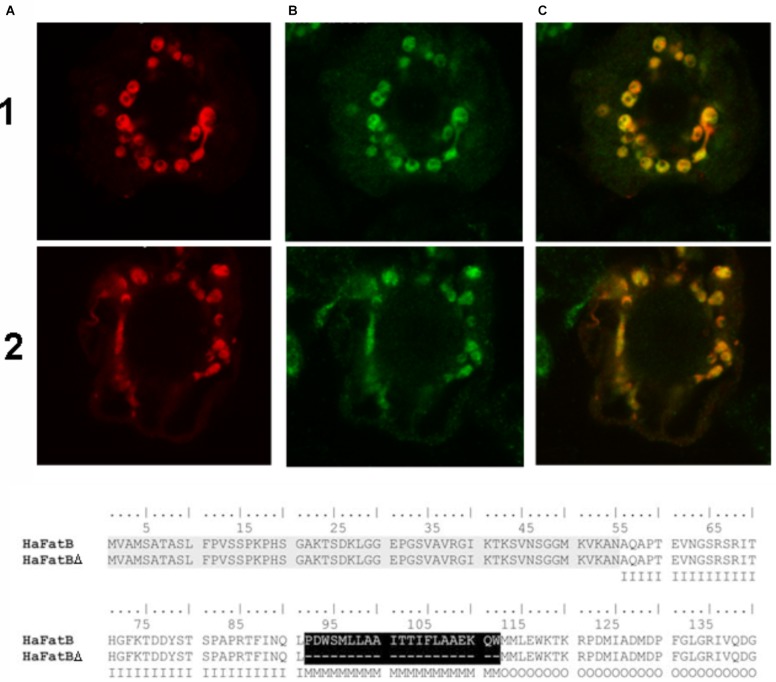
Representative confocal laser scanning microscopy images images of Cherry-tagged *Ha*FatB-Cherry or *Ha*FatBΔ Cherry expressed transiently (via biolistic bombardment) in BY-2 tobacco cells. Images in column **(A)** show FatB-Cherry row **(1)** and FatBΔ-Cherry row **(2)**, while those in column **(B)** represent fluorescent Alexa 488 dye-conjugated to Anti-N-acetyl glutamate kinase (Anti NAGK). The corresponding merged images of all the cells are shown in column **(C)**. The amino acid alignment of sequences corresponding to *Ha*FatB and the mutant *Ha*FatBΔ, highlighting the hydrophobic region removed in black. This hydrophobic region was localized using different bioinformatics programs and it was removed by PCR splicing using the sequence of *Ha*FatB. The first 50 amino acids correspond to the signal peptide: I, Inside amino acid; M, transmembrane helix amino acid; O, outside amino acid.

Given that *Ha*FatB, but not *Ha*FATA, possesses a potential membrane-binding domain (**Figure 3**), it is possible that the protein is localized not only in the soluble stroma, but also at a stromal-facing membrane(s), namely the inner envelope membrane and/or thylakoid membrane. To test this possibility, we examined whether removal of the hydrophobic sequence from *Ha*FATB influenced its subplastidial localization in BY-2 cells. Thus, this domain was removed from *Ha*FATB by PCR according to alignment in **Figure 8**, producing the gene *Ha*FATBΔ. As shown in **Figures 8-1A–C,2A–C**, both *Ha*FATB and *Ha*FATBΔ appended to the Cherry fluorescent protein colocalized with endogenous NAGK, similar to *Ha*FATB-GFP (and *Ha*FATA-GFP) (**Figures 7-1A–C,2A–C**).

**FIGURE 9:**
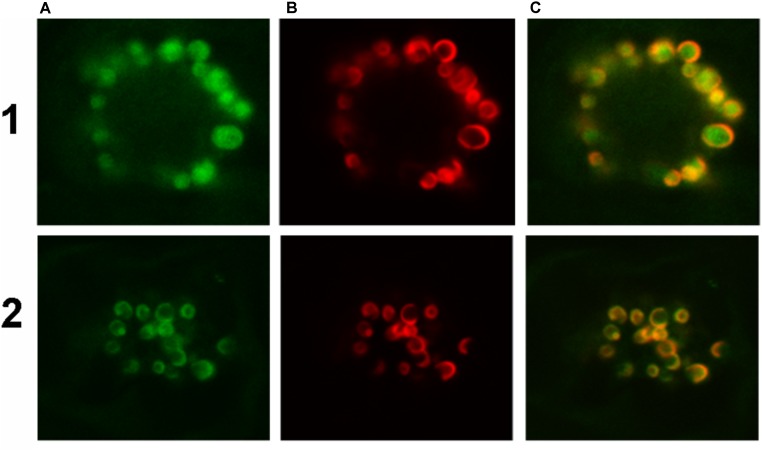
Representative confocal laser scanning microscopy images of GFP-tagged *Ha*FatA or *Ha*FatB expressed transiently (via biolistic bombardment) in BY-2 tobacco cells. Images in column **(A)** show FatA-GFP row **(1)** and FatB-GFP row **(2)**, while those in column **(B)** represent the expression of sub-cellular marker proteins co-expressed in the same cells, specifically the inner chloroplast envelope protein Tic40-RFP. Corresponding merged images of all the cells are shown in column **(C)**.

The latter data support the conclusion that *Ha*FatB is localized to the soluble stroma, like *Ha*FATA, and does so in the absence of its hydrophobic sequence. Nevertheless, the results from additional experiments involving co-expressed TIC40-RFP, which consists of the 40-kDa subunit of the translocon at the inner envelope membrane of chloroplasts fused to the red fluorescent protein (Dhanoa et al., 2010; Breuers et al., 2012), revealed that the plastid localization of *Ha*FATB can sometimes vary. Thus, as shown in **Figures 9-1A–1C**, transiently expressed *Ha*FATA-GFP displayed a fluorescence pattern that was delineated by the characteristic, cresent-like fluorescence pattern attributable to co-expressed TIC40-RFP. This is attributable to the ectopic expression of TIC40 causing a proliferation and remodeling of the inner envelope membrane (Breuers et al., 2012; Machettira et al., 2012) and served here to help differentiate protein localization at the inner membrane versus stroma. By contrast, *Ha*FatB-GFP readily co-localized with co-expressed TIC40-RFP (**Figures 9-2A–C**), indicating that *Ha*FATB, unlike its counterpart, is associated with the inner envelope membrane in plant cells. Taken together, these subcellular location studies confirm that both sunflower thiosterases target to plastids, but that *Ha*FATA and *Ha*FATB differ with regards to their localization to the stroma or stroma and/or inner envelope membrane, respectively.

### Isolation of the Subcellular Plastid Fraction and Western Blotting

To further assess the subplastidal localization of *Ha*FatB, the localization of the enzyme was analzyed using a complementary *in vitro* plastid purification-based strategy. More specficially, we isolated plastids from developing sunflower seeds (15 days after fertilization) and lysed them, separating the soluble phase from the membranes by ultracentrifugation. Proteins from both fractions were then analyzed by Western blotting with anti-*Arabidopsis (At)* FatB antibodies (Dörmann et al., 1995), the specificity of which was confirmed by detecting recombinant (His)_6_-tagged *Ha*FatB in a soluble fraction from *E. coli*. As controls, the distribution of PFP enzyme activity, which is a cytosolic protein (Burrell et al., 1994; Schaffer and Petreikov, 1997) and LACS, which is located in the plastid envelope (Aznar-Moreno et al., 2014), were assessed to confirm the purity of the isolated soluble and membrane plastid fractions.

**FIGURE 10:**
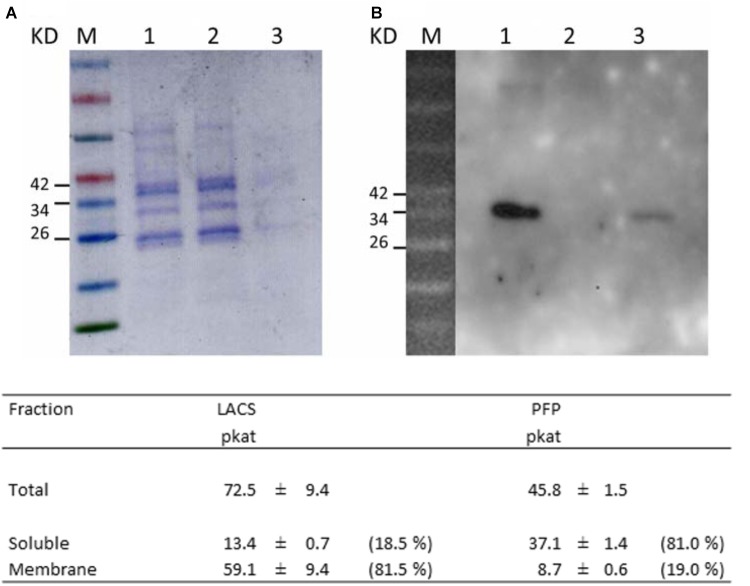
Subcellular localization of *Ha*FatB in sunflower seed plastids. **(A)** SDS–PAGE protein gel stained with Coomassie blue of: the molecular weight markers (line M), total plastid protein (lane 1), soluble subcellular fraction (lane 2), and membrane fraction (lane 3). **(B)** Western blot probed with anti-*At*FatB antibodies showed that *Ha*FatB was in the total and membrane fractions. Results from biochemical assays to determinate the purity of the soluble and membrane fractions. LACS (long chain acyl-coA synthetase, membrane fraction control) in the total, soluble and membrane fractions of plastids was assayed to determine the purity of the soluble fraction. Pyrophosphate-dependent phosphofructokinase (PFP, soluble fraction control) in the total, soluble and membrane fraction of plastids was assessed to determine the purity of the membrane fraction. Data corresponded to the average of three determinations from different biological replicates plus minus SD.

As shown in **Figure 10**, the majority (81.5%) of LACS activity, PFP activity (81%) and oleoyl-ACP thioesterase (not shown) was found in the membrane and soluble fractions, confirming the suitable separation of these plastid fractions and the purity of the preparation for western blot assays (**Figure 10**).

When western blots of the soluble and membrane fractions were probed with the anti-*At*FatB antibody, a protein band of the expected size was detected in both the total and membrane fractions, but not in the soluble fraction (**Figure 10**). Hence, it appears that *Ha*FatB localizes predominantly to plastid membranes of developing sunflower seeds, while the distribution of the protein in plastids in BY-2 cells appears to be influenced by its ectopic over-expression, including when co-expressed with other plastid proteins, namely TIC40 (**Figure 9**). Unfortunately, the western blot record does not give notice of the role of the hydrophobic domain within *Ha*FatB. The different location of these two enzymes probably influences in their interaction with the other enzymes involved on plastidial fatty acid synthesis, which are soluble and located in stroma and enforces the hypothesis of these enzymes having different roles in plant lipid synthesis.

## Conclusion

The FatA and FatB enzymes are essential to maintain the necessary *de novo* fatty acid flux from plastids to the endoplasmic reticulum. Based on its expression and the profile of fatty acid accumulation, FatA is the dominant thioesterase during the period of oil accumulation in sunflower seeds. The novel information available on the 3D structure of plant thioesterases allowed the *Ha*FatA and *Ha*FatB binding pockets to be modeled, indicating that the kinetic and structural differences of both enzymes are driven mainly by the residues that determine the interaction with the acyl moiety of the substrate, independent of the interaction with the ACP or the catalytic residues. Confocal microscopy showed that *Ha*FatA is a soluble protein that is translocated to the stroma of the chloroplast in BY-2 tobacco cells. By contrast, the distribution of *Ha*FatB was far less clear and it was affected by the experimental design. These results do not allow us to conclude whether this protein is localized in the stroma or membrane anchored. Nevertheless, the data obtained from the stromal and membrane plastidial fractions of sunflower seeds strongly suggest that the *Ha*FatB protein is removal anchored to the membrane through its hydrophobic region. While we cannot be sure if the protein anchors to the thylakoid membrane or to the inner membrane of the plastid, it is clear that both thioesterases differ in their fatty acid selectivity and localization. These differences probably reflect how differential interactions with the soluble plastidial FAS I and FAS II, and soluble acyl-ACP desaturase, affect the export of the *de novo* synthesized fatty acids in sunflower seeds, supporting the hypothesis of a different role of these enzymes within plant lipid metabolism.

## Author Contributions

JA-M performed most of the experimental work. RS and AM-P participated in plasmid construction and rtPCR. SG and RM participated in localization experiments. MVC, EM-F, RG, RM, and JS participated in the work direction and experimental design. JS, EM-F, and RM wrote and revised the manuscript.

## Conflict of Interest Statement

The authors declare that the research was conducted in the absence of any commercial or financial relationships that could be construed as a potential conflict of interest.

## Footnotes

1 http://www.enzyme.cbirc.iastate.edu/

2 http://www.expasy.org/spdbv/

3 http://zhanglab.ccmb.med.umich.edu/I-TASSER/

4 http://www.cbs.dtu.dk/services/ChloroP/

5 http://www.cbs.dtu.dk/services/SignalP/
